# Fig abscission as a defense mechanism of *Ficus* trees against parasitism by non-pollinating fig wasps

**DOI:** 10.1038/s41598-025-86135-3

**Published:** 2025-01-14

**Authors:** Shang-Yang Lin, Bruno Di Giusto, Anthony Bain

**Affiliations:** 1 Institute of Ecology and Evolutionary Biology, National Taiwan University, Taipei, Taiwan; 2 Research Center of Sleep Medicine, Taipei Medical University, Taipei, Taiwan; 3 Journalism and Mass Communication Program, International College, Ming Chuan University, Taipei, Taiwan; 4 Department of Biological Sciences, National Sun Yat-sen University, Kao-hsiung, Taiwan; 5 International Ph.D. Program for Science, National Sun Yat-Sen University, Kao-hsiung, Taiwan

**Keywords:** Selective abortion, *Ficus*, Non-pollinating fig wasp, Plant defense, Evolution, Ecology, Evolutionary ecology

## Abstract

How does the fig tree *Ficus benguetensis* protect its investment in the production of figs and pollinating fig wasps against parasitism from non-pollinating fig wasps? This study documents a previously overlooked defense mechanism: fig abscission—the natural shedding of the fig fruit as a defense mechanism. Our bagging experiments showed that both the absence of pollination and high parasitism levels lead to the abortion of *F. benguetensis* figs, with positive correlations between parasitism levels, increased abscission rates, and decreased pollinator production. Moreover, we found that high parasitism corresponds to shortened fig development periods until abscission, while medium parasitism levels result in fewer pollinators. Our findings suggest that abscission may function as a resource conservation strategy, as most of the tree’s investment in the figs occurs post-pollination. This study uncovers for the first time the use of fig abscission as a unique defense against non-pollinating fig wasp parasites, broadening our understanding of plant defense mechanisms within mutualistic interactions.

## Introduction

Understanding the mechanisms by which plants protect themselves against herbivores remains a key component of research in ecology and agronomy^1^. Plants exhibit many defense strategies to deter herbivores from consuming their tissues^2,3^. Mechanical defenses, such as hairs, thorns, and spines^4,5^, are the most conspicuous barriers against herbivores, but most plant defenses involve chemical compounds that act as deterrents or toxins^3^. These defenses can be constitutive (i.e., permanently present) or induced (i.e., produced as a response to herbivore attacks). Additionally, plant defenses may be direct (e.g., the secretion of toxic compounds that repel herbivorous insects) or indirect (e.g., the plant releases substances to attract the herbivores’ natural enemies, such as ants^6,7^). The interplay between these defenses determines the overall resilience of plants against herbivory^1^. However, despite the diversity and complexity of plant defenses, herbivory continues to inflict damage on plants. Many herbivores, especially plant specialists, remain unaffected by plant toxins as they evolve innovative ways to circumvent plant protective measures^8^.

One such method is galling. Galls are plant tissues coerced into gall formation in response to insect larvae or other organisms^9,10^. These structures provide a safe environment for developing insects, shielding them from predators and environmental stresses^11^. They also provide a nutrient-rich food source for the developing insect, enabling rapid growth^11^. Finally, gall resources are often exclusive to insects, reducing competition for food and space^11^. This unique way of hijacking resources may leave the plant with little choice but to sacrifice one of its organs. Furthermore, variations in the presence and abundance of natural enemies^7^ mean that neither direct nor indirect defense strategies are always effective. As a result, heavily attacked plants may resort to a more drastic strategy by sacrificing their own tissues to deter herbivores.

This is done through organ abscission, whereby a plant allows an organ to shed and fall by triggering cell death in the abscission zone of the organ^12^. This shedding is a complex and adaptive process that allows plants to respond to changing environmental conditions, conserve resources, and optimize their growth and reproduction strategies^13,14^. While leaf abscission is the most common form^15^, flower abscission^16^ and fruit abscission^17,18^ have also been observed. In a recent study of the peach tree, *Prunus persica*, researchers observed leaf abscission as a response to gall formation by the peach aphid, *Tuberocephalus momonis*: Infested leaves were shed significantly earlier than healthy ones, effectively limiting the success of the parasite^19^. Other studies have shown that abscission significantly reduces the survival rate of galling insects in leaves^20,21^ and in fruits^17,18^. These practical observations underline the adaptive significance of organ abscission as a defensive response against galling herbivores.

While galling is a common strategy used by many insects to coerce plant tissues into creating a secure environment for their offspring, the obligate pollination mutualism between pollinating fig wasps (Hymenoptera: Agaonidae) and *Ficus* trees represents a unique example of this phenomenon. Unlike most galling insects, these wasps not only induce gall formation but also play a critical role as the exclusive pollen vectors of fig trees. Female fig wasps, carrying the pollen from their natal figs, enter a receptive fig through a minute opening called the ostiole. Inside, they distribute the pollen across the stigmas of the individual flowers within the fig and deposit their eggs within the fig ovules. After hatching, the larvae thrive on the fig ovule and the specially produced galling tissue, ultimately emerging as adults to carry the pollen to a new fig. In this nursery pollination mutualism, pollination services are reciprocated with safe oviposition sites and nourishing environments for larval development^22^.

In fig-fig wasp mutualism, fig abscission may seem counterintuitive, as it leads to the loss of both fig ovules (seeds) and pollinators. However, in some American *Ficus* species, a unique situation arises where pollinators occasionally lay eggs without delivering pollen to the Fig^23^. In response to this lack of pollination, fig trees exhibit specific physiological reactions: They reduce resource allocation to unpollinated figs, which consequently produces fewer and smaller wasp offspring^24^. Some species go even further by aborting unpollinated figs entirely, thereby minimizing the cost of developing seedless Fig^23^. This response, however, is not universal across all *Ficus* species; for instance, in *Ficus microcarpa*, the presence of cheating wasps (relatives of the pollinating wasp) does not lead to increased abscission rates^25^. The prevalence and variability of these defensive mechanisms among other fig species remain largely unexplored, and whether fig trees employ similar strategies to defend against other threats, such as herbivores or parasites, is still in its early stages^26^.Adding another layer of intricacy to their mutualistic relationships, fig trees engage in complex interactions with parasitic wasps. Also called non-pollinating fig wasps (NPFWs), these parasitic wasps lay eggs from outside the figs using their long ovipositors and all have a larval phase within the Fig^27^. They can be parasitic to fig ovules, pollinating wasps, and other NPFW species^27^. Found in nearly all *Ficus* species worldwide^27–29^, NPFWs impose significant costs on *Ficus* by reducing seed production and pollen transfer as they often kill the larvae of the pollinating wasps^30^. This complex interaction between parasites and mutualistic wasps within the plant raises an evolutionary dilemma for *Ficus* trees: How can *Ficus* reduce the cost of NPFW parasitism without harming their mutualistic partners?

One clue to how fig trees cope with NPFW parasitism lies in the way NPFWs lay their eggs. Unlike fig pollinators that enter figs to lay eggs, most NPFWs lay their eggs from outside. They stab their ovipositor through the fig wall, the tissue forming the fig structure. This fig wall might seem mostly ineffective against specialist parasites like NPFWs^31^, but it may expose them to predator attacks by increasing ovipositing time^32^. Studies show that ants often patrol fig trees^33,34^, potentially reducing NPFW oviposition rates^35,36^. However, the success of this biotic defense varies^7^. The absence of ants in some trees^34^ and the failure to attract effective ant species in deterring herbivores^37^ can weaken the defense. In such scenarios, fig abscission might serve as a final defense, eliminating a parasitized fig that, though pollinated, will not lead to the survival of the pollinators. By aborting such a fig, the tree prevents a unnecessary investment in a fruit that cannot fulfill its role in producing pollen vectors.

Interestingly, figs have been observed falling from trees during their early development, particularly after pollination, in some *Ficus* species^38,39^, including *Ficus benguetensis*^40^. This fig tree is gynodioecious but functionally dioecious, meaning male and female functional organs are found on separate plants, and the fig wasp *Ceratosolen wui*^41^ ensures the pollen transfer from male to female trees. Several species of NPFWs are associated with *Ceratosolen wui* larvae developing in the male Fig^29^, though whether they kill the pollinator larvae and parasitize the fig ovules directly or parasitize the developing pollinator larvae within these ovules remains to be determined. In both cases, the parasitized PFW larvae die. Uniquely, *Ficus benguetensis* is one of the only fig species known to secrete extrafloral nectar on its Fig^42^, potentially optimizing ant patrolling during vulnerable stages of fig development^43^. However, this indirect biotic defense seems insufficient to deter NPFWs that commonly appear at the surface of its figs.

We propose that *F. benguetensis* may be employing fig abscission, an existing mechanism typically used for removing unpollinated figs, in a novel way as a defensive response triggered by NPFW parasitism. This potential repurposing of abscission could represent an intriguing example of how plants may adapt existing traits to address new ecological challenges. We then sought to investigate two main questions related to fig defense against NPFWs: 1) Is organ abscission in *Ficus benguetensis* a defense against parasites?2) If so, how frequently is it employed, and under what conditions? To answer these questions, we conducted bagging experiments to compare fig abscission in response to varying levels of parasitism on *Ficus benguetensis* figs in Taipei, Taiwan. Our research brings new insights into the adaptative strategies that allow plants to defend their resources and mutualistic partners against parasitism.

## Methods

### Study species

*Ficus benguetensis* Merril 1905 (Subgenus *Sycomorus*, Section *Sycocarpus*) is found in East Pacific Asia: Ryukyu Islands (Japan), Taiwan, and the Philippines^44^. Reaching up to 12 m, trees mainly grow in humid environments. This *Ficus* species is gynodioecious, exhibiting both hermaphroditic individuals (having both male and female reproductive organs) and female-only individuals. The figs are located on the trunk or apical branches^43^. Figs on hermaphrodite trees (hereafter called male figs) contain stamens and ovaries with short-styled pistils, which produce pollen and pollinating fig wasps. These figs play a critical role in maintaining the pollinator population.

On the other hand, female figs are characterized by ovaries with long-styled pistils that exclusively produce seed. The extended length of these female styles functions as a mechanical barrier, preventing pollinating wasps from laying their eggs.

**Fig. 1:**
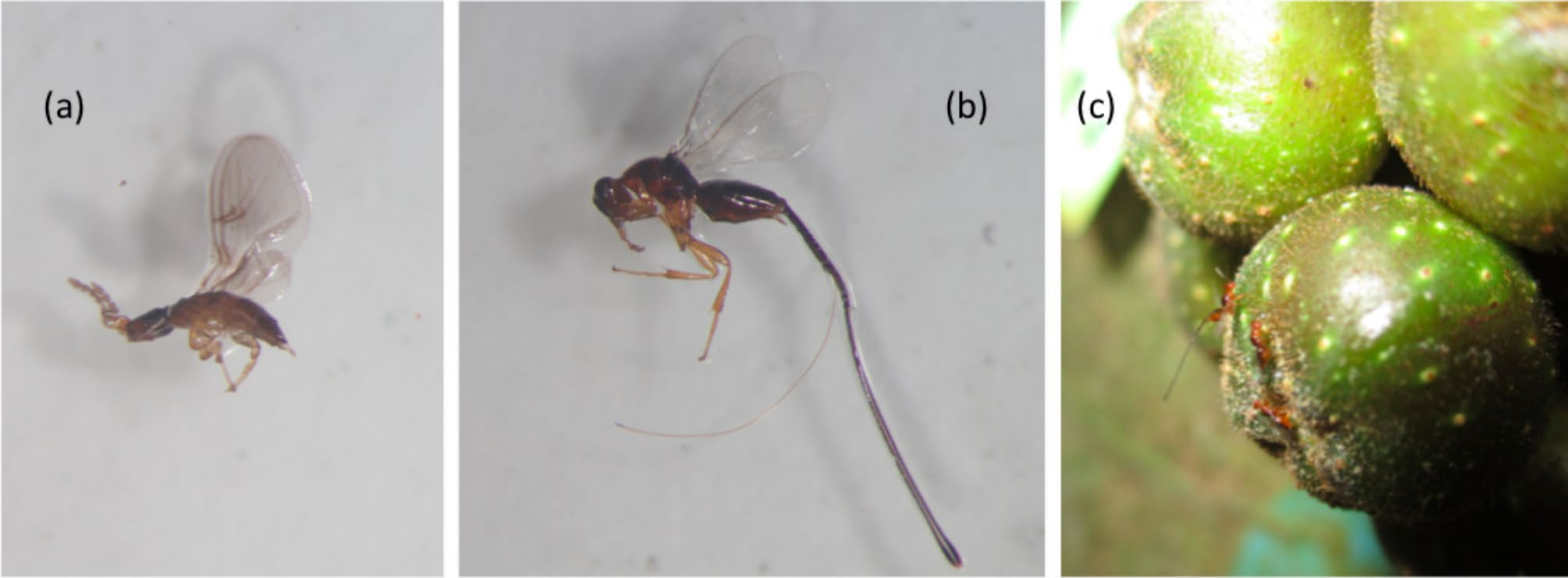
The wasp fauna associated with *Ficus benguetensis* (only female wasps are displayed): (a) the pollinating wasp *Ceratosolen wui*, (b) the parasitizing wasp *Philotrypesis taida*, and (c) three parasitizing wasps *Philotrypesis* sp. gathered on a fig recently pollinated by *C. wui*.

In Taiwan, the *F. benguetensis* pollinating wasp is *Ceratosolen wui*^39^; Fig. 1a). Additionally, at least four non-pollinating fig wasp (NPFW) species are associated with *F. benguetensis and C. wui*: the recently described *Philotrypesis taida* (Fig. 1b) and *Sycorycteridea taipeiensis*^45^, and two other Sycoryctinae wasp species^29^. Generally, *Philotrypesis* spp. and most Sycoryctinae wasps are parasitoids, whose larvae feed on other wasp larvae^46,47^. Sycoryctine wasps do not enter the figs; they rather penetrate the fig wall using their long ovipositors to lay eggs in ovules already oviposited by pollinating wasps. Once their larvae hatch, they either consume the host larvae directly (parasitoid) or feed on the gall endosperm and starve the host larvae (kleptoparasite)^48^. However, information on the studied species is yet to be published, and their feeding regime is still uncertain.

The experiments done on plants and insects (which are not endangered) followed the relevant guidelines of the IUCN.

### Phenological cycle of *Ficus benguetensis*

The phenological cycle of the male *Ficus benguetensis* figs is marked by specific physiological transitions and ecological events across five distinct developmental phases (Table 1).

**Table 1:** Summary of the developmental phases in the phenological cycle of male figs of *F. Benguetensis*.

Phase	Description	Duration
Premature Phase (Phase A)	Initiation and early development of the fig before female flower receptivity	Four weeks
Receptive Phase (Phase B)	Female flowers are receptive and the fig is attractive to fig wasps	A few hours to a few days
	Pollinators enter male receptive figs, oviposit in the ovules inducing tissue growth to form the gall	
Inter-floral Phase (Phase C)	The fig grows	Six to eight weeks
	The pollinator larvae feed on the plant tissues in the fig’s development	
	The NPFW *Philotrypesis taida* are observed on the figs shortly after phase B	
Emergence Phase (Phase D) [only for male figs]	Apterous wasp males emerge first and mate with females inside their natal galls	After wasp emergence, either the fig drops or rots away (Lin pers. obs.)
	After mating, females collect pollen and exit the fig	
	If the pollinating wasps have been parasitized, they die, and non-pollinating wasps emerge in their stead	
Ripening Phase (Phase E) [only for female figs]	Ripening of the fig. The fig becomes attractive to frugivores	A few days to two weeks

The described duration for each phase is derived from Lin^40^.

### Wasp-introduction experiment

The experiment was conducted from June to September 2013 in Fu-Yang Eco-Park, Taipei city (121°33’25.74”, 25°1’0.97”) where only two NPFW species, *Philotrypesis taida* (Fig. 1b) and *Sycorycteridea taipeiensis*, were observed. *Philotrypesis* wasps were much more common, so this species was selected for the wasp-introduction experiment.

Five *F. benguetensis* trees were chosen for this experiment, which involved bagging figs with a 12-cm^2^ mesh bag to prevent unwanted wasps from laying eggs. Then, a controlled number of fig wasps was introduced in each bag containing a receptive fig. Wasps needed for the experiment were collected on the figs of neighboring trees using a pooter and then released into a mesh bag surrounding a fig. The experiment included five different treatments:

1. One pollinator (positive control, *N* = 15).
2. One pollinator and one *Philotrypesis* wasp (low parasitism rate, *N* = 13).
3. One pollinator and three *Philotrypesis* wasps (medium parasitism rate, *N* = 12).
4. One pollinator and five *Philotrypesis* wasps (high parasitism rate, *N* = 5).
5. One *Philotrypesis* wasp (negative control, *N* = 10).

Pollinating wasps were placed on the fig ostiole and observed until entering. Then, NPFWs were placed directly on the surface of the fig and secured in the mesh bags. Treatment 5 served as a negative control in two ways: first, to test whether *Philotrypesis* wasps alone could sustain fig development, and second, to validate our bagging method by confirming that no pollinating wasps could enter the bagged figs unintentionally.

To minimize tree-specific effects and ensure the robustness of our findings, a cross-over design was employed in our experiment, allowing each of the five *Ficus benguetensis* trees to experience every treatment. Wasp introduction dates were recorded, and the fig trees were surveyed bi-weekly to determine insect emergence or fig abortion dates. After wasp emergence, all wasp offspring were collected, identified, and counted.

### Fig growth survey

In this study, 77 figs were randomly harvested from five male *F. benguetensis* trees located in Fu-Yang Ecology Park, focusing on figs from the trunks to ensure consistency, with seven classified as pre-receptive and 70 as post-receptive, enabling a detailed examination of resource allocation before and after the pollination phase. Each fig was measured, dried, and weighed to establish a relationship between its diameter and dry weight. We hypothesized that the fig tree would invest minimal resources in fig growth before pollination while increasing investment after pollination to support gall and wasp development. We employed various regression models (linear, exponential, quadratic, simple cubic, and full cubic) to fit the scatterplot and selected the best-fitting model to characterize this relationship. This methodology enabled us to evaluate resource allocation by the fig tree across different developmental stages and estimate potential resource savings from aborting parasitized figs.

### Statistical analysis

Statistical analyses of the wasp-introduction experiment were conducted using Fisher exact tests, Kruskal-Wallis H tests, and Mann-Whitney U tests, performed with the software Systat 12 (Systat Software, Inc., Point Richmond, California, www.systat.com).

To evaluate the differences in growth between the pre- and post-receptive phases, we utilized bootstrapping methods. A total of 1,000 bootstrapping samples were generated by resampling the original dataset with replacement. Linear regression models were then fitted to each resampled dataset, and the slopes and intercepts were recorded. This approach allowed us to estimate 95% confidence intervals for the slopes and intercepts of the linear regression lines for each phase.

## Results

The impact of various levels of pollination and parasitism on wasp production and fig development in *F. benguetensis* was observed through five experimental treatments, each varying in the number of pollinators and parasitic *Philotrypesis taida* wasps involved.

### Abscission, wasp production, and parasitism

Of the 55 bags initially set up on the trees, 48 (87.3%) were retrieved and seven were lost. Table 2 illustrates the effects of the treatments on wasp emergence and fig development.

**Table 2:** Wasp emergence and fig developmental success under different treatments.

Treatment	1	2	3	4	5
	No parasitism (*n* = 9)	Low parasitism (*n* = 13)	Medium parasitism (*n* = 12)	High parasitism (*n* = 5)	No pollination (*n* = 9)
Figs reaching D-phase	8^a^	12^a^	7^a^	0^b^	0^b^
Abscised figs	1	1	5	5	9
Fig development					
Days to D-phase	47.5 ± 6.26^a^	42.1 ± 2.89^a^	57.3 ± 12.63^a^	NA	NA
Days to abscission	31	30	28.6 ± 3.83^a^	17.6 ± 1.69^b^	28.4 ± 1.36^a^
Wasp production					
Total wasp number	143.5 ± 25.24^a^	141.17 ± 21.76^a^	70.3 ± 20.38^b^	NA	NA
Pollinator: *C. wui*	143.5 ± 25.24 ^a^	103.8 ± 18.82 ^a^	47.6 ± 17.44 ^b^	NA	NA
NPFW: *P. taida*	0	37.3 ± 6.62 ^a^	22.7 ± 4.21 ^b^	NA	NA
NPFW proportion	0	24.9%	40%	NA	NA

Treatment 1—one *Ceratosolen wui* pollinator, no *philotrypesis* wasp (positive control); treatment 2—one pollinator and one *philotrypesis* wasp (low parasitism rate); treatment 3—one pollinator and three *philotrypesis* wasps (medium parasitism rate); treatment 4—one pollinator and five *philotrypesis* wasps (high parasitism rate); treatment 5—no pollinator, one *philotrypesis* wasp (negative control). Mean ± standard error is provided when applicable.

^a b^ Different letters indicate statistically significant differences (*P* ≤ 0.05); Fisher exact tests were used to compare the number of abscised figs reaching the D phase; all other tests are Kruskal-Wallis and Mann-Whitney tests. NA: If none of the figs reached the D phase, NA is attributed to this case.

**Fig. 2:**
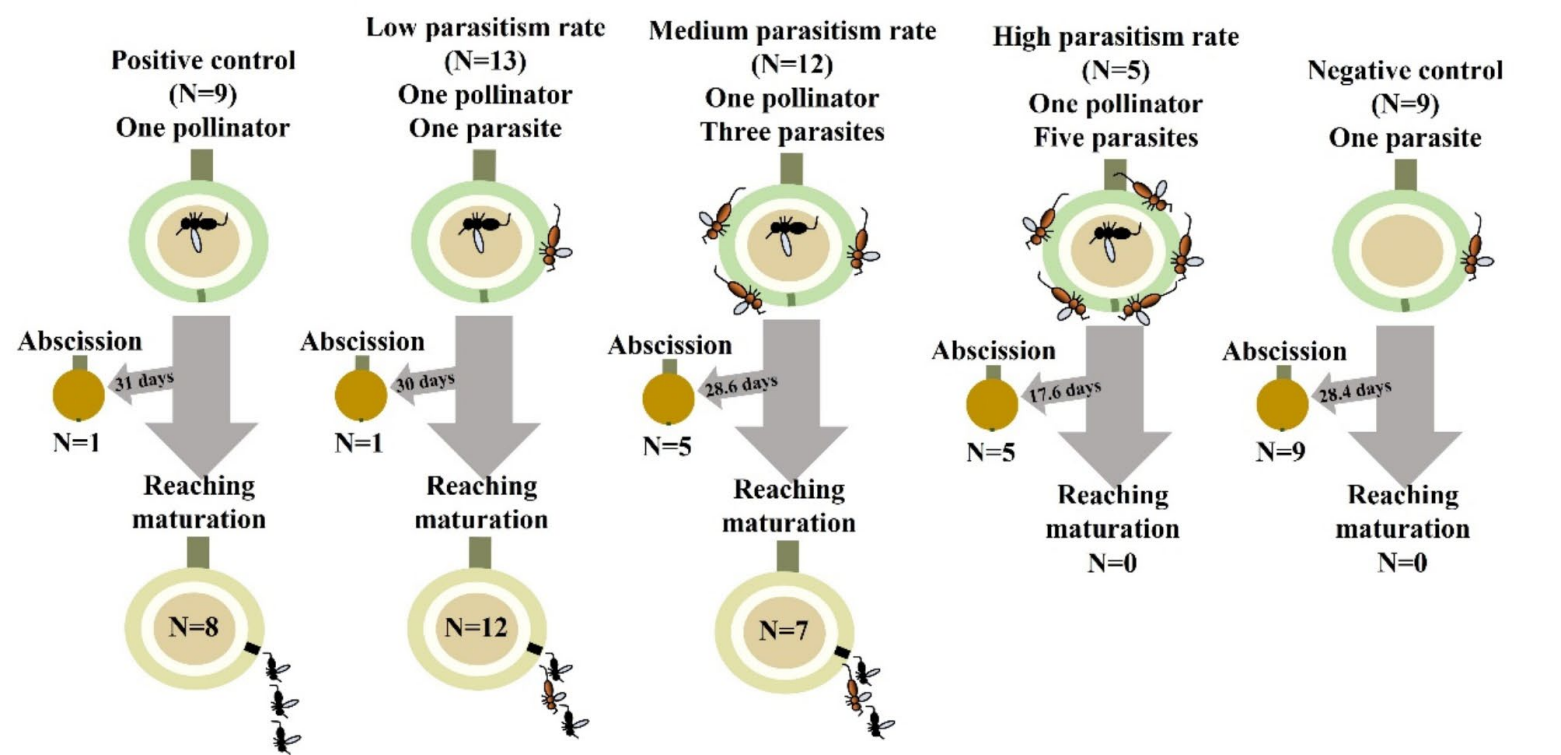
Diagram summarizing the various treatments of the bagging experiment.

Most of the figs in treatments 1 and 2 reached the wasp-releasing stage (Phase D), while only about half did so in treatment 3, and none in treatments 4 and 5 (Table 2; Fig. 2). High parasitism rates and absence of pollination were associated with the abscission of all the figs under these treatments.

The duration of the development between the wasp introduction (pollination) to phase D (wasp-releasing phase) did not vary significantly between treatments 1, 2, and 3 (Kruskal-Wallis test, *p* = 0.787, Table 2). However, the period from wasp introduction to abscission was significantly shorter for treatment 4 (high parasitism) compared with treatments 3 (medium parasitism) and 5 (no pollination) (Kruskal-Wallis test, *p* = 0.001, post hoc test: treatments 3 vs. 4: *p* = 0.006; treatments 3 vs. 5: *p* = 0.96; treatment 4 vs. 5: *p* = 0.0027).

Wasp production, encompassing both pollinators and NPFWs, varied significantly among treatments. Specifically, treatment 3 (medium parasitism) showed a significantly lower number of wasps than treatments 1 and 2 (Kruskal-Wallis test, *p* = 0.008). A similar pattern was observed when only considering the pollinator *C. wui*, with no significant difference in the number of emerged pollinating wasps between treatments 1 and 2, but a marked decrease in treatment 3 (Kruskal-Wallis test, *p* = 0.005; posthoc tests: treatment 1 vs. 2: *p* = 0.099; treatment 1 vs. 3: *p* = 0.0009; treatment 2 vs. 3: *p* = 0.027). Finally, we found that the number of emerged NPFWs was significantly higher in treatment 2 (low parasitism) compared to treatment 3 (medium parasitism) (Mann-Whitney, *p* = 0.0286). As low parasitism treatment also saw approximately 75% of emerged wasps as pollinating wasps, compared to 60% in treatment 3 (medium parasitism), we conclude that a higher level of parasitism reduced both the pollinating and non-pollinating wasp numbers. These findings underscore the complexity of wasp production dynamics under varying ecological pressures.

Statistical analyses indicated no significant tree effects on fig abscission or retention (Fisher’s exact test two-sided *p* = 0.91), total wasp emergence (Kruskal-Wallis test p-values: 0.41, 0.66, and 0.05 for treatments 1, 2, and 3, respectively), or retention time (Kruskal-Wallis test p-values: 0.24, 0.72, 0.07, 0.67, and 0.19 for treatments 1 to 5, respectively), demonstrating consistency across trees within each treatment.

### Fig size and investment

After comparing several regression models (linear, exponential, quadratic, simple cubic, and full cubic), we found that quadratic (R^2^ = 0.5638) and full cubic models (R^2^ = 0.5639) provided the best fits. Following the principle of parsimony, we selected the quadratic model. Further, log-log analysis yielded a slope of 2.86 [95% CI: 2.54, 3.18], falling between what would be expected for a pure surface area relationship (slope = 2) and a pure volume relationship (slope = 3). This suggests a more complex growth pattern than either simple model would predict, possibly reflecting changes in tissue density during fig.

**Fig. 3:**
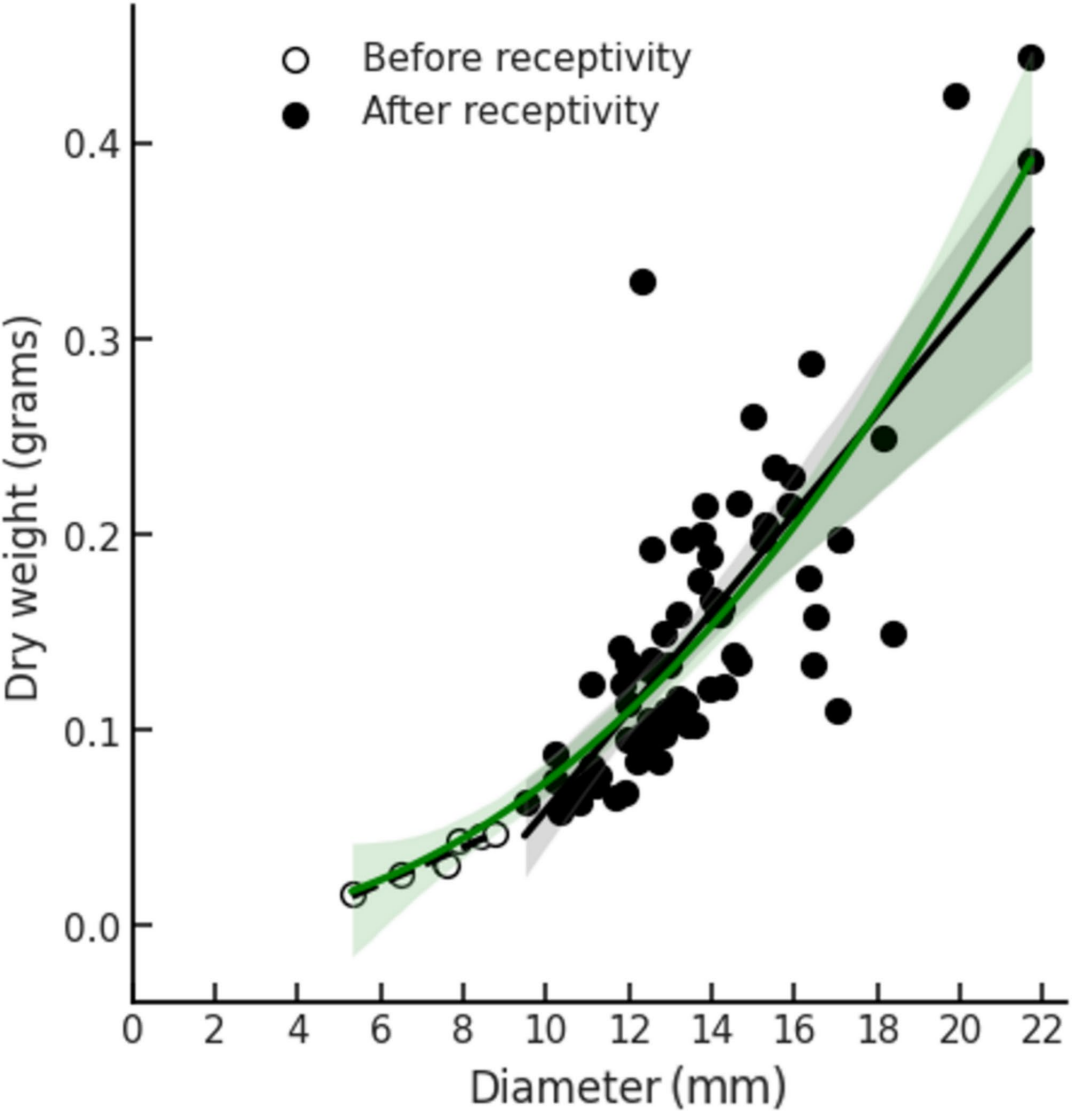
Relationship between the diameter (in millimeters) and the dry weight (in grams) of 77 figs. The data are fitted with the quadratic model *Y = 0.0009 × 2 − 0.0023x + 0.0037*, which exhibits a strong goodness-of-fit with a coefficient of determination (*r*^*2*^) of 0.98 and a highly significant p-value of. 2.7 × 10 − 54. The white circles ○ represent figs in the premature phase (before receptivity) and the black circles ● represent figs in the interfloral phase (after receptivity). To further illustrate the accelerating rate in fig growth during the later stages of development, we provide two linear regression lines: a dashed line for figs before receptivity (y = 0.01x – 0.03, *r* = 0.96, *p* < 0.001) and a solid line for figs after receptivity (y = 0.03x – 0.19, *r* = 0.79, *p* < 0.001).

The selected quadratic model indicates that fig diameter reliably predicts dry weight and reveals varying resource allocation patterns across fig development stages (Fig. 3)., The 95% confidence intervals for the slopes and intercepts for the phase A figs (Slope: [0.0069, 0.0140]; Intercept: [– 0.0736, – 0.0199]) and for the phase C figs (Slope: [0.0185,0.0306]; Intercept: [– 0.2621, – 0.1058]) did not overlap, indicating significantly different growth patterns between phases. While these findings demonstrate a substantial resource investment after pollination, our sampling approach cannot determine its precise timing during fig development.

In summary, our data point to complex patterns in how fig trees manage resources during fig development, demonstrating a clear shift in investment strategy pre- and post-pollination. The bootstrapping results support this conclusion through statistically significant differences in the slopes and intercepts of the regression lines for the two phases. These findings corroborate our earlier observations that fig trees effectively conserve resources by rapidly aborting heavily parasitized figs.

## Discussion

This study elucidates key dynamics in the mutualism between the pollinating fig wasp *Ceratosolen wui* and the fig tree *Ficus benguetensis* when facing a parasite, the non-pollinating fig wasp, *Philotrypesis taida*. Using bagging experiments to compare fig abscission in response to varying levels of parasitism, our study first showed that both a high level of parasitism and the absence of pollination resulted in the abscission of the figs of *F. benguetensis*. Second, the results demonstrate that increased levels of parasitism induced an increased abscission rate and a reduced production of pollinating and non-pollinating wasps. Third, we document that high levels of parasitism were associated with shorter periods of fig development until abscission. Fourth, the findings also reveal that medium levels of parasitism were connected to lower numbers of pollinators. Finally, the study supports the idea that abscission might serve as a mechanism for conserving *Ficus* resources, as most of the tree material investment in the figs occurs after pollination.

In our study of *Ficus benguetensis*, we found that unpollinated figs could persist for up to four weeks post-receptivity before abscission, a trait consistent with the adaptive strategy of *Ficus* species to extend receptivity under unpollinated conditions^48,49^. This observation indicates the tree’s capacity to maintain figs for a potential extended window for pollination, though not necessarily extending the receptive phase itself. Such extended persistence may represent a bet-hedging strategy (i.e., a strategy of reducing risk by diversifying reproductive strategies, ensuring survival and reproductive success to cope with environmental uncertainties) during periods of low pollinator availability (i.e., the winter season for some *Ficus* species^39^). Conversely, our data indicates that *F. benguetensis* rapidly abscises figs under heavy parasitism within an average of 17 days, suggesting an active physiological defense mechanism that limits resource investment in figs with compromised offspring. This rapid abscission in response to parasitism highlights the ability of fig trees to prioritize resource investment in figs with a higher likelihood of successful pollinating wasp production.

Further, we observed a large resource investment in *F. benguetensis* figs following successful pollination or possibly oviposition by *C. wui*, a pattern similar to the one observed in the male figs of another dioecious species, *F. fulva*^50^. This post-pollination weight increase indicates a strategic metabolic investment to support the development of the pollinating wasp offspring. We also found an increased variability in weight among larger figs. The complex growth pattern between fig diameter and dry weight suggested by the log-log slope of 2.86 possibly involves changes in tissue density during development. This variation likely reflects the different reproductive outcomes among figs, as each fig produces different numbers of pollinator offspring and supports varying levels of NPFW parasitism. If the dry weight of the fig is a reliable measure of reproductive investment, this suggests that *F. benguetensis* can adjust its resource allocation, possibly as a countermeasure to the impact of parasitism by NPFWs. Such a result will be consistent with the Selective Resource Allocation model in monoecious fig trees proposed by Jander & Herre^24^, which posits that *Ficus* trees allocate more resources to better-pollinated figs. Considering our focus on the parasitism of NPFWs on pollinating fig wasps, which is exclusive to male figs, future studies should investigate if female figs possess comparable abscission and resource allocation mechanisms to protect against seed parasitism, thereby preserving their reproductive output.

In our study of the dioecious *Ficus benguetensis*, we observed a significant reduction in pollinating and non-pollinating wasp productions with increased levels of parasitism by NPFWs, a pattern that diverges from findings in other fig species. Research on monoecious figs like *Ficus andicola*, *F. sur*, and *F. vallis-choudae* by Cardona et al.^30^ and Kerdelhué & Rasplus^27^ showed that parasitoid NPFWs primarily impacted the male function (pollen dispersal) by reducing the pollinator populations through parasitizing or competing for oviposition sites. Galler NPFWs, in contrast, had variable impacts on both male and female function. However, in *F. benguetensis*, a dioecious species where parasitoids do not influence female figs (pollinating wasp larvae being unable to develop in female figs), we observed a unique scenario. Heavy parasitism led to a drastic reduction in pollinating *and* non-pollinating wasp productions and complete abscission of figs, indicating a distinct defensive strategy focused on male function. This also contrasts with the findings of Weiblen et al.^51^ in *F. trachypison*, a dioecious fig tree where a dynamic equilibrium was observed. Parasitism by NPFWs never exceeded 10% per fig and was inversely related to pollinator density, suggesting a stabilizing effect on the mutualistic relationship. In *F. benguetensis*, the decline in both pollinator and NPFW productions with increased parasitism and the shedding of the most heavily parasitized figs suggests that intense parasitism often resulted in the premature death of both the host and the parasites, a phenomenon common in parasitoid wasps^52^. This pattern of fig abscission, differing markedly from the balanced interactions reported by Weiblen et al.^51^, could significantly alter the dynamics between pollinating and non-pollinating wasp populations, impacting the balance between mutualism and parasitism in this system.

**Fig. 4:**
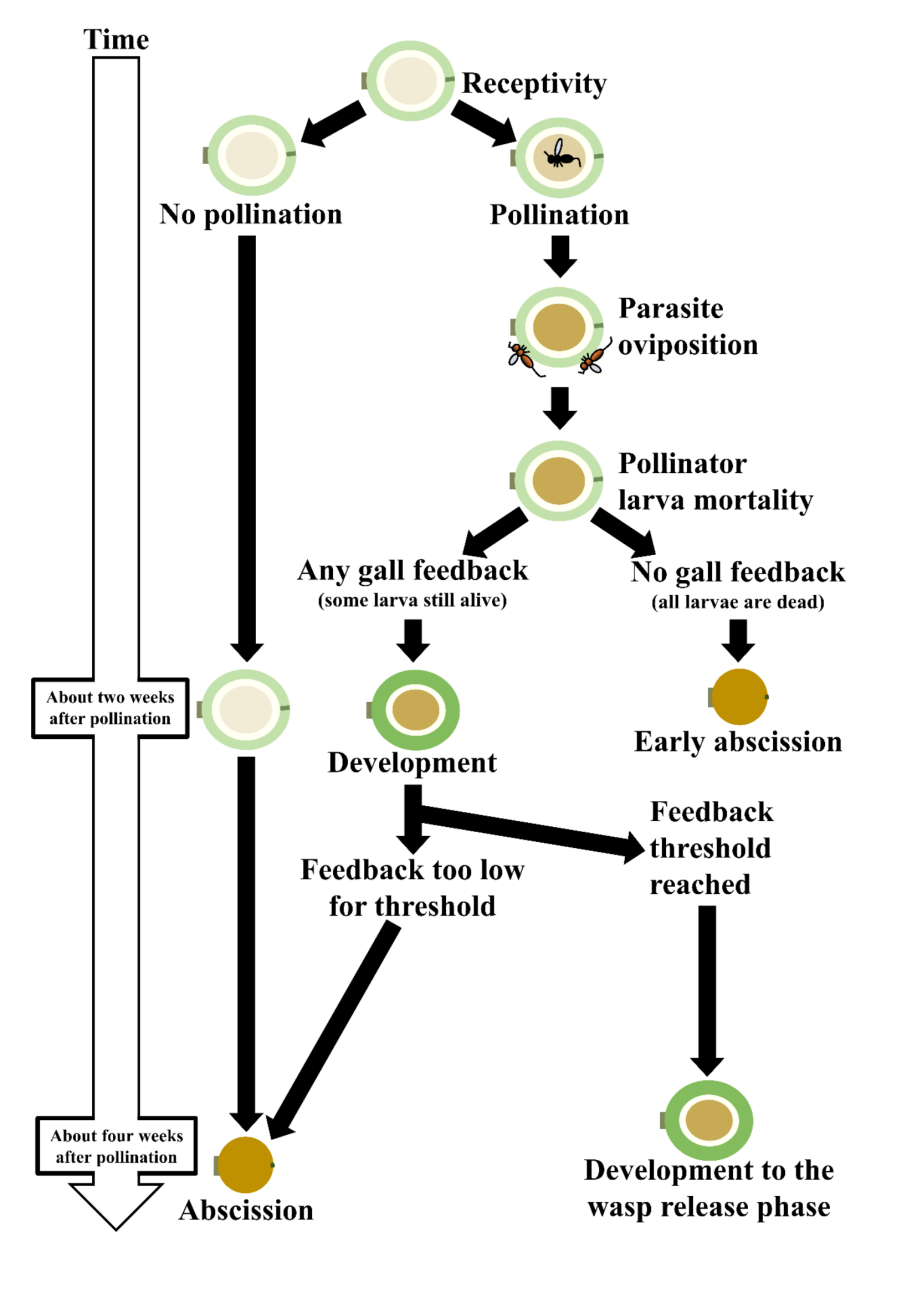
Summary of the abscission defense mechanism in *Ficus benguetensis*.

This, however, raises the question of how *Ficus* trees determine which figs to abscise (Fig. 4). We propose two hypotheses:

**Detection of NPFW Larvae by fig trees**: *Philotrypesis taida* is a likely parasitoid species targeting *Ceratosolen wui* larvae. In fig wasp research, feeding habits are often inferred based on the timing of wasp arrival at Figs^52,53^. Early arriving wasps (before pollinators) are typically gallers, while late-arriving wasps (after pollination) are usually parasitoids. *Philotrypesis* species, including *P. taida*, consistently arrive after pollination, strongly suggesting a parasitoid lifestyle. This is further supported by our experimental results. In Treatment 5, where only *P. taida* was introduced without pollinators, all figs were abscised. This indicates that *P. taida* cannot induce gall formation or sustain fig development on its own, consistent with a parasitoid lifestyle. The fact that figs only develop when both pollinators and *P. taida* are present (Treatments 2–4) further supports the idea that *P. taida* relies on pollinator larvae for its own development.

Parasitoid fig wasps typically lay eggs on pollinating wasp larvae, and their larvae feed on the hosts as they develop^54^. The fact that *P. taida* lay eggs shortly after *C. wui* oviposition and the figs are aborted several weeks later suggest a strategy where the parasitoid allows its host to grow and develop after parasitism, feeding on it over time (i.e., koinobiont parasitoid). However, without direct observation of larval development, we cannot conclusively determine whether *P. taida* is a kleptoparasite or a parasitoid.

Very little is known about the fig tree defense mechanisms against such parasitoids. Indeed, the current scientific understanding of these host-parasite dynamics is limited, with very few well-documented cases of host defenses against parasitoids^55^. While there is more extensive knowledge about gall insects, especially oak wasps^56^, understanding their parasitoids remains significantly underexplored^57^. In such context, we hypothesize that the fig tree might detect changes within the fig environment. While the number of oviposition scars could potentially serve as a signal for NPFW presence, this is unlikely to be the main factor in our experiment as we used natural, moderate levels of NPFW introduction that typically allow successful fig development in field conditions. Instead, we propose that parasitoid larvae may emit secretions or that parasitized pollinator larvae may produce altered secretions. If these secretions reach a certain threshold, the fig tree may react by abscising the affected fig, a response that could be a critical aspect of its defense strategy.

**Detection of pollinating Wasp Gall signals by fig trees**: Here, we propose that fig trees respond to signals from pollinating wasp galls, leading to fig abscission if these signals are interrupted. Our study, which assumed all pollinating wasps carried pollen, showed a 100% abscission rate associated with high parasitism levels, as opposed to approximately 40% with medium parasitism levels. This high abscission rate, despite the presence of pollinators, suggests that factors beyond pollination influence abscission. It thus offers new insights compared to the previous understanding that primarily associated fig abscission with defense against cheating pollinators^24,58,59^.

Two main points support the reasoning for this hypothesis: first, factors other than pollination likely trigger fig development in dioecious fig trees; second, galls are known to form an interface between the host plant and the galling insect.

For the former trait, research on Panamanian *Ficus* species, which are monoecious, showed that Panamanian trees allocate fewer resources to or abscise figs cheated by pollinators^24,59^. Indeed, as the cheating pollinators carry little or no pollen, unpollinated or poorly pollinated figs will have no or a subpar production of seeds. However, even in monoecious species, the absence of pollination might not be the sole explanation for fig abscission. Research by Wang et al.^50^ on *Ficus racemosa*, a monoecious species from the same subgenus as *F. benguetensis*, revealed that the rate of fig abortion decreased with an increase in the number of pollinating wasps found in the figs. This study concluded that the occurrence of fig abortion depends on the number of pollinators and the presence of pollen. This suggests that for the subgenus Sycomorus, factors other than pollination may be necessary for the continued development of figs. We should expect a different scenario to unfold with *F. benguetensis*, a dioecious species that develops seedless male figs. In this species, male figs exclusively contain galls. Thus, factors enhancing pollinator production and male fig reproductive fitness, such as pollinating wasp oviposition and gall formations are more likely to trigger fig development.

Once the egg hatches, the pollinating wasp larva coerces the fig tissue into forming a gall. The gall modifies the plant physiology around itself, redirecting plant resources toward the gall and insect growth^59,61–66^. This implies that the galls signal the fig for resource allocation. This phenomenon exists in *F. citrifolia* in Brazil where galling NPFWs can prevent the abortion of 41% of the Fig^67^. We propose that a reduction in the intensity of the signal sent below a certain threshold, or its cessation, could trigger the fig abscission. The variability in the length of fig development and abscission rates observed in our study could be attributed to differences in the number of galls produced by pollinating wasps and the extent of oviposition by NPFWs affecting the number of wasps produced per Fig^68^. For instance, Zhang et al. (2020) demonstrated that parasitoid presence can lead to increased mortality of pollinator larvae beyond direct replacement. They found that for each parasitoid produced, approximately 1.9 pollinator larvae were killed, which could further impact the signaling dynamics within the Fig^69^. This complex interaction between pollinators, parasitoids, and the fig itself may contribute to the variability we observed in fig development and abscission rates. Further research is essential to understand the specific signals and thresholds that lead to fig abscission in dioecious fig species. As the mechanisms behind developing these “seedless fruits” remain undetermined^70,71^, further investigation could warrant critical insights into the mechanisms allowing the passage between monoecy and dioecy.

The ecological and evolutionary implications of fig abscission as a defense strategy in the fig-fig wasp mutualism are complex. First, fig abscission as an anti-herbivore mechanism is critical to the coevolutionary relationship between fig trees and fig wasps. Indeed, the fig tree ability to abscise figs in response to parasitism can influence the evolution of wasp behaviors and strategies, potentially leading to an evolutionary arms race between the tree, its pollinators, and parasitic wasps. Second, by selectively aborting figs with high levels of parasitism, the tree can indirectly control the population of non-pollinating wasps, which may help maintain the balance between mutualistic and parasitic interactions, thus contributing to the stability of the fig-fig wasp mutualism. Third, fig abscission affects the population dynamics of both pollinating and non-pollinating fig wasps. By delaying the growth of figs under medium parasitism and quickly abscising figs that are heavily parasitized, the fig tree can more efficiently allocate its resources to figs with a higher likelihood of successful pollinator development. This selective investment ensures that the tree resources are not wasted on figs unlikely to contribute to its reproductive success. Finally, the defensive role of fig abscission could be considered an exaptation - a trait that has been co-opted for a new function different from its original purpose. While fig abscission likely evolved initially for other functions such as resource conservation during environmental stress or removal of unfertilized reproductive structures, it has been adapted here as an effective defense mechanism against parasitic wasps. This exemplifies how existing physiological mechanisms can be repurposed through evolution to serve new functions.

While our study offers valuable insights, it has limitations. Although constrained by the challenges of fig-wasp bagging experiments, our sample size achieved statistical significance. Our crossover experimental design controlled for tree-specific effects and temporal variations, ensuring that any external factors would likely affect all treatments equally. The clear treatment-specific effects we observed suggest that our experimental manipulations, rather than external factors, were the primary drivers of the observed differences. Future work could further validate and expand our findings through larger-scale studies across varied seasons and habitats. Subsequent research could explore the physiological mechanisms underlying fig abscission, including chemical signaling between figs and wasps, and investigate broader ecological consequences within the fig-wasp community. Additionally, examining the relationship between fig size, parasitism, and reproductive fitness in male figs, as well as studying the growth and defense strategies of female figs in *F. benguetensis*, would provide a more comprehensive understanding of this species reproductive ecology.

.

## Conclusion

This study extends the current understanding of mutualism, parasitism, and coevolutionary dynamics, particularly in the interactions between *Ficus benguetensis*,* Ceratosolen wui*, and *Philotrypesis taida*. For the first time, it is reported that a *Ficus* species can strongly decrease the number of NPFWs using fig abscission as a defensive mechanism to minimize resource loss. The research highlights the complexity of mutualistic relationships when influenced by parasitic pressures. Fig abscission as a defense strategy has significant implications for the ecology and evolution of fig trees and their associated wasps. It influences the dynamics of mutualistic and parasitic interactions, resource allocation, and population control, likely playing a vital role in the stability and functioning of this mutualism. Our study offers a more nuanced view of these complex ecological interactions and suggests that traditional models of mutualism may require revision to better consider the roles of third-party species.

## Data Availability

All data generated or analyzed during this study are included in this published article.
